# An Investigation into the Relationship between Density and Pulverization Ratio for Tannin-Furanic Foam

**DOI:** 10.3390/ma16196384

**Published:** 2023-09-24

**Authors:** Wenbin Yuan, Hisham Essawy, Qiaomei Ding, Xiaojian Zhou, Xinyi Chen

**Affiliations:** 1 Yunnan Provincial Key Laboratory of Wood Adhesives and Glued Products, Southwest Forestry University, Kunming 650224, China; wenbinyuarn@hotmail.com (W.Y.); dqm2169242115@163.com (Q.D.); 2 Department of Polymers and Pigments, National Research Centre, Cairo 12622, Egypt; hishamessawy@yahoo.com

**Keywords:** formaldehyde, glutaraldehyde, tannin, foam, pulverization ratio

## Abstract

Four types of classical tannin-based foam samples were prepared via different methods in the current study with an attempt to find out the impact of each one on the physico-mechanical properties. The results of performed tests showed similarity to the general trend of related research, with typical negative relation between the foam density and mechanical strength. A critical point was found for each type of foam samples, for example, for tannin-formaldehyde foams (TFF), they were in the range of 85–95 kg/m^3^, while for tannin-glutaraldehyde foams (TGF), mechanically-generated tannin foams (MTF) and steam-driven tannin furanic foams (STDF), they were about 52–62 kg/m^3^, 53–63 kg/m^3^, and 73–83 kg/m^3^, respectively. This implies a significant change for density and mechanical strength, has been dig out by intensive experimental results and analysis. In addition, a non-liner relationship between density and pulverization ratio was obtained by fitting the curves obtained by the experiment results. Finally, visualization using scanning electron microscopy (SEM) together with evaluation of the compression strength presented a deeper insight to illustrate the different factors affecting foam density and pulverization ratio.

## 1. Introduction

Tannin-furanic based rigid foams (TF) exhibit very promising development prospects as a new type of biomass foams because of their excellent properties, including light weight, flame-retardancy, thermal insulation, high natural contents, sustainability, etc. Condensed tannin is a natural product based on flavonoid units, showing a complex chemical structure with multimer, together with different types of oligomers, and so displaying variation in reactivity [1,2]. The most original formulation of TF appeared in the 1970s, but this foam product did not demonstrate good performance [3]. Meikleham and Pizzi [4] have collaborated since 1994 to develop a new formulation, which was based on the co-reaction of a condensed tannin with furfuryl alcohol. However, the use of formaldehyde was necessary to ensure stability of the foam during preparation. In the years since then, especially recently, considerable attention has been paid by many researchers to achieve a common goal, which was the eventual obtaining of an environmentally friendly tannin-based rigid foam product with a good combination of properties. Thus, several efforts have been put into this product towards gradual improvement of its properties, while attempting to optimize the foam formulations to incorporate high green contents [5]. For example, formaldehyde is well known as a potentially carcinogenic material, therefore glyoxal and glutaraldehyde were utilized instead so that the resulting product became environmentally friendly [6]. Interestingly, both aldehydes achieved good results, giving rise to another competitive advantage for the promotion of tannin-based foams.

Despite the development of TF showing a favorable trend, there was a very serious problem that plagued many research groups, which was its high pulverization ratio. The basic properties of different types of TF were evaluated, in which it was evident that there were significant differences between due to tannin’s characteristics [7]. It is well known that the activities of tannin resources are highly related to their flavonoid unit types and quantity [8]. This can influence the reaction rate and the extent to which tannin is liable to interact with other active sites, which finally affected the reaction results and degree of cross-linking. It can be presumed that this is probably a crucial condition that affects the pulverization ratio of TF. In addition, speculation has been made as to whether the cross-linking or density of the TF would have an effect on the pulverization ratio. More experiments were also elaborated recently to verify this point.

Some research groups focused their works on finding out a way to reduce the phenomenon of high pulverization ratio in the case of TF [9,10]. Initially, starting with internal cross-linking, a cellular/nonporous TF was prepared via the addition of extra biomass [11]. Lignin, as a biomass resource abundantly stored in nature, was added to the tannin-based foams [12]. Wu et al. [13] found that the density of the as-obtained TF foam generally increased with reduction in the cell size of tannin foam. In another study, it was attempted to further adjust the pulverization ratio of TF by an environmentally friendly cross-linker to totally substitute formaldehyde with plant protein, which was found to be an efficient strategy [14]. It was mainly performed on the active sites in the tannin molecules via combination with the free radicals in the amino acids of the protein to form a good network of contacts in order to promote cross-linking. These methods largely enhanced the mechanical performance. One of the most intuitive manifestations is that the pulverization ratio for the modified TF was reduced efficiently, but inevitably the density of TF was increased in some formulations. In addition, vegetable oils were considered, and a significant reduction in the dropout of tannin-based foams could be achieved by the addition of palm oil [10,15]. Another option was to reconsider TF from a structural point of view and design the terminal foam product. The sandwich structure, as an innovative aspect for practical application, was successfully applied to ameliorate the defects of TF [16]. This method played an important role in reducing the pulverization ratio of TF foam as an engineering method for surface enhancement.

However, the most attractive among these methods for developing the mechanical properties of TF foam was the upgrading of the starting material and preparation method to achieve the desired purpose. The blowing agent, as one of the commonly utilized starting materials for traditional TF foam preparation, had a non-negligible effect on the density. The influence of the two options can be quickly detected by varying its addition and abandoning it altogether [17,18]. Santiago-Medina et al. reported a mechanical stirring foaming method, which introduced some air into the resin during the vigorous stirring process, forming a core of air bubbles in the precursor resin, resulting in the obtaining of a new type of TF foam [19]. Moreover, another novel method was suggested, namely steam-driven foaming, and it was reported that this showed a lower pulverization ratio that reached more than 60% compared to traditional TF foam [20]. Ultimately, these methods, whatever updates the feed-stock or reported novel preparation strategies were carried out, were explained as an efficiency pathway to upgrade the properties of TF foam, especially the pulverization ratio.

Further, the high pulverization ratio is a very serious problem of tannin-based foams, which can influence the mechanical performance. Several studies have reported on the relation between foam density and its mechanical performance, with a unified conclusion that the mechanical performance decreases significantly when the foam density is below a certain range. Therefore, this point or density range (inflection point) can serve as an important index for foam preparation in future work, especially for considering the field of application and optimization of the manufacturing formulation.

Herein, we adopted the preparation of several types of tannin foams via different foaming methods, including the traditional foaming method, mechanical stirring, and steam-driven foaming, and the prepared foams are subject to intensive investigations. Different amounts of foaming agent (diethyl ether) were added, or water, as the main ingredient to regulate the foam density according to the specialty of the preparation. To the best of our knowledge, this is the first detailed systematic investigation dedicated to exploring the impact of various important factors on the pulverization ratio. For all studied formulations, the control variables method was used to ensure the reliability of the experiments. SEM and compression strength were analyzed to obtain deeper insight into the relations between cell morphology, density, pulverization ratio, and compression strength.

## 2. Materials and Methods

### 2.1. Materials

Commercial mimosa condensed tannin extract (Acacia mearnsii) was purchased from TANAC S/A (R. Torbjorn Weibul, Montenegro, Brazil). Furfuryl alcohol (AR, C_5_H_6_O_2_, 98%) was a product of Shanghai Macklin Biochemical Co., Ltd. (Shanghai, China). Formaldehyde (AR), Tween-80 (CP) and p-toluene-sulfonic acid (p-TSA, AR, ≥98.5%) were purchased from Sinopharm Chemical Reagent Co., Ltd. (Shanghai, China). Glutaraldehyde (AR 50%) was purchased from Chengdu Kelong Chemical Co., Ltd. (Chengdu, China). Formaldehyde and diethyl ether (AR) were supplied by Yanglin Industrial Development Zone Shandian Pharmaceutical Point Company. (Kunming, China). The distilled water utilized in all experiments was prepared in the laboratory. All other chemicals were used without any further purification.

### 2.2. Foam Preparation

The anticipated foam formulations are shown in Table 1. The tannin-formaldehyde foams (TFF), tannin-glutaraldehyde foams (TGF), and mechanically-generated tannin foams (MTF) were prepared as follows. First, tannin was weighed and placed into a beaker, labeled as B. Then, furfuryl alcohol mixed with different aldehydes (formaldehyde or glutaraldehyde), Tween-80, and diethyl ether in turn, was marked as A. Thereafter, mixture A was poured into the tannin material B while vigorous stirring was conducted for 3–5 min to form a homogeneous mixture. Then, 15 g of 65% aqueous solution of p-TSA (freshly prepared by dissolution in distilled water) was added and stirred with a glass rod until the color of the resin turned from brown to black. In the case of MTF, the diethyl ether was removed and formaldehyde served as the cross-linker. The foaming precursor was whipped by a household electric mixer (HM-955, the maximum rotational speed can reach 2500 r/min) to obtain a liquid foam. Finally, the soft or liquid foams were put into an oven for 4 h at 80 °C to obtain a rigid foam sample, which was stored for at least 24 h at room temperature before testing.

On the other hand, the preparation process of steam-driven tannin furanic foams (STDF) was undertaken as follows. Briefly, 30 g of bark concentrated solution was taken and put into a plastic cup. Then, another solution comprising 10 g of furfuryl alcohol mixed with 1.5 g of Tween 80 and 10 g distilled water was added into the plastic cup and stirring was performed vigorously using a mechanical stirrer for 3–5 min to obtain a homogeneous resin. Afterward, 15 g of p-TSA solution was added slowly under constant stirring for 2 min to ensure uniform mixing, named as foaming resin. Finally, the foaming resin was poured into a special filter paper box and quickly placed in a steam environment (100 °C on a water bath to provide constant steam). The foaming process continued until the liquid resin solution was fully converted to foam by the incorporation of air under sealed steam conditions. The STDF was taken out after 20 min, and transferred into an oven for curing and drying at 80 °C for 4 h, then stored for at least 24 h at room temperature before testing.

### 2.3. Apparent Density

The apparent density was evaluated by following the method reported in the literature [20]. The appropriate specimen size was set at ((5 ± 0.5) cm × (5 ± 0.5) cm × (5 ± 0.5) cm), and the calculation of the apparent density (kg/m^3^) was accomplished according to Equation (1):

(1) $$\rho =\frac{m}{v}\times 10^{6}$$

where *m* is the weight of foam sample; *v* represents the volume of foam sample. The average density was obtained as an average of five repeated determinations.

### 2.4. Pulverization Ratio

**Figure 1:**
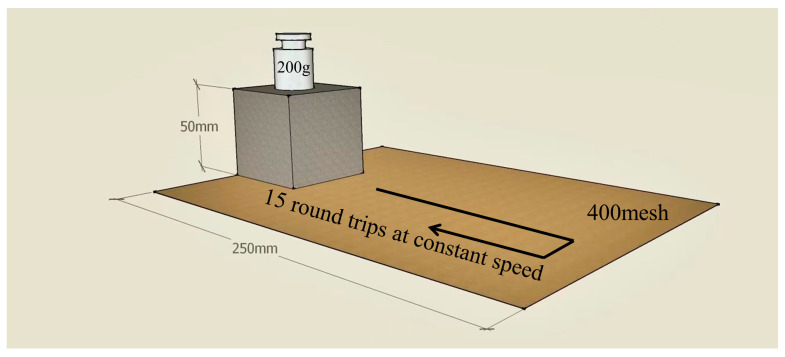
Graphical design for the determination of pulverization ratio.

The pulverization ratio of the foam was obtained by using the relevant literature of international standard. The middle part of the finished foam was cut into cubes of (5 ± 0.5) cm × (5 ± 0.5) cm × (5 ± 0.5) cm. The pulverization was performed in the transverse direction (the transverse mode was parallel to the bottom). The pulverization ratio was tested on the same surface of the foam (Figure 1). One surface of the sample was placed on sandpaper of 400 mesh with a length of 250 mm, then 200 g weight was placed on its upper surface. The foam was pulled 15 times at a horizontal uniform speed and the remaining mass of the sample was recorded. The pulverization ratio was calculated according to Equation (2):

(2) $$M_{f}=\frac{m_{1}-m_{0}}{m_{1}}\times 100\%$$

where $m_{1}$ is the original mass of the specimen (g); $m_{0}$ is the total mass of the sample after the test (g); $M_{f}$ is the pulverization ratio (%), then the average value of three tests for each sample was considered.

### 2.5. Scanning Electron Microscopy Investigation (SEM)

The morphology of the foam was observed at a voltage of 15 kV at different magnifications using a ZEISS Gemini 300 scanning electron microscope (ZEISS Group, Jena, Germany). The prepared samples were made into a cross-section and sprayed with gold, then inserted into the equipment. SEM micrographs of modified tannin foams were acquired at 100× and 400× magnifications.

### 2.6. Mechanical Strength

The mechanical strength in terms of compression was evaluated by using a universal testing machine (AG-50KN, Shimadzu, Kyoto, Japan) loaded with a 50 kN head, according to the Chinese National Standard GB/T8813-2020 [22]. The samples were cut into specimens with dimensions of 30 × 30 × 1.5 mm^3^ to evaluate the compression strength of the cellular foam layer. The tests were conducted at a constant loading rate of 2.0 mm/min, and the final compression strength was defined as the maximum force before 15% of the foam was deformed, divided by the compressed surface area. The compression strength curves were the average of five replicate determinations.

## 3. Results and Discussion

**Figure 2:**
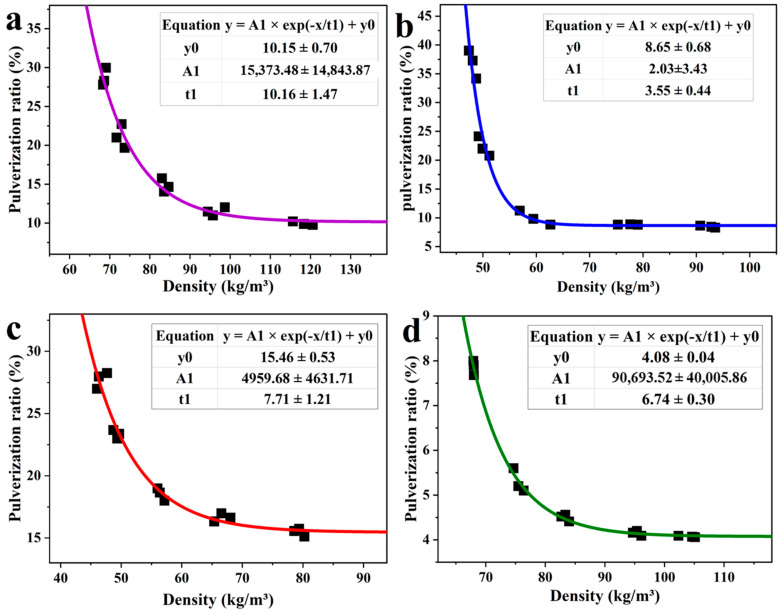
Trend graph between density and pulverization ratio of the foams. (**a**) TFF; (**b**) TGF; (**c**) MTF; (**d**) STDF.

Traditional tannin furanic foams were prepared normally with different types of aldehydes as cross-linker. Herein, two kinds of traditional tannin-furanic foams were prepared the following relevant literature by using formaldehyde and glutaraldehyde as cross-linker, respectively [4,6]. The density of the resultant foam was controlled by varying the dosage of the blowing agent in order to comprehensively investigate the relationship between foam density and pulverization ratio. Furthermore, two additional types of tannin-furanic foams were prepared, via foaming induced by mechanical stirring, (MTF) and steam-driven foaming, (STDF), for further verification. The difference in the amount of water significantly affected the density of the foam in the absence of physical or chemical blowing agents. This factor was very helpful in the preparation of our foam samples. The four basic TF formulations were repeated, with the only difference that of the foam sequence compared to previous experiments. The determination of density and pulverization ratio was carried out and the corresponding results are shown in Figure 2, where it can be seen that the densities of all foam types are in the range of 40–120 kg/m^3^, which presents a systematic range of the basic data for TF. A transformation of cross-linker from the most commonly utilized formaldehyde to glutaraldehyde was followed by the innovative approaches of mechanical stirring and the steam-driven method, which enabled comparison and study of the correlations of the patterns they present.

Diethyl ether, as the main blowing agent, played a key point in the foaming process of TFF and TGF [17,23]. The foam density for these kinds of foam product decreases along with the rise in the added amount of foaming agent. This result is similar to the literature reports, and is mainly caused by the volume of tannin furanic resin expanding under the driving force of the evolving blowing agent. The precursor resin gave off a lot of heat under the self-condensation reaction of furfuryl alcohol (FA), which promoted volatilization of the foaming agent [18]. The foam cells will continue to grow when the amount of foaming agent is large enough, so that the foam density can be controlled easily by adjusting the addition of blowing agent.

TF has a significant disadvantage concerning its high brittleness, which is a shortcoming that cannot be ignored in most rigid foams [24,25]. The pulverization ratio, which is an internationally accepted indicator, was considered as a parameter in this study for evaluating the brittleness of TF foam [21]. It can be noticed that, as the density of TFF and TGF decreases, the corresponding pulverization ratio gradually increases (Figure 2a,b). Moreover, a critical node can be seen from these curves, at around 80–90 kg/m^3^ for TFF and 52–60 kg/m^3^ for TGF, indicating that the pulverization ratio will rise sharply while the foam density goes below the node. Inversely, the gradient of pulverization ratio for those two foam samples will be located in a limited range while the foam density is higher than the node, ultimately arriving at a plateau. This phenomenon suggested an inflection point, i.e., node, existing for the trend between density and pulverization ratio. It could be found via a large number of experiments that the density of TFF was inversely related to the pulverization ratio. This is an encouraging result that could be used to forecast the application field based on the relationship between foam density and its pulverization ratio, normally the mechanical performance of these kinds of traditional foams.

A non-linear equation which shows the relationship between the density and the pulverization ratio of these foams was fitted from the experiment investigation. This non-linear equation will be very helpful in future experiments to predict the change in pulverization ratio for this kind of foam when the density is varied. It is speculated that there are two main reasons for this inverse relationship between density and pulverization ratio. One is the pore size for the foam, which gradually increases as the density decreases, resulting in a limited number of intact cell structures in the unit volume. The entity quality of foam in the unit volume will also decrease. The increase in cell size associated with the drop in foam density is another reason. In addition, the thinning of the cell wall under progressive conditions affects the overall performance of TF foam. Therefore, all types of crosslinkers can finally reveal a similar trend for the relationship between foam density and pulverization ratio.

To further investigate the relationship between foam density and pulverization ratio, the tannin-based foams were prepared via different approaches, by mechanical stirring (MTF) and by a steam-driven (STDF) approach. Foams with different densities were successfully prepared by adjusting the amount of added water [21]. The pulverization ratio was also evaluated, and the resulting data are represented in Figure 2c,d. There is a very significant inverse relationship between the density of the foam and the pulverization ratio, with an interesting inflection point. The corresponding graphs for density and pulverization ratio, as well as the positions of the inflection points, provide a good reference for these trends, as well as for the preparation of the relevant types of foam in the future, which allows us to find the ideal type of foam for a given application in a straightforward manner.

**Figure 3:**
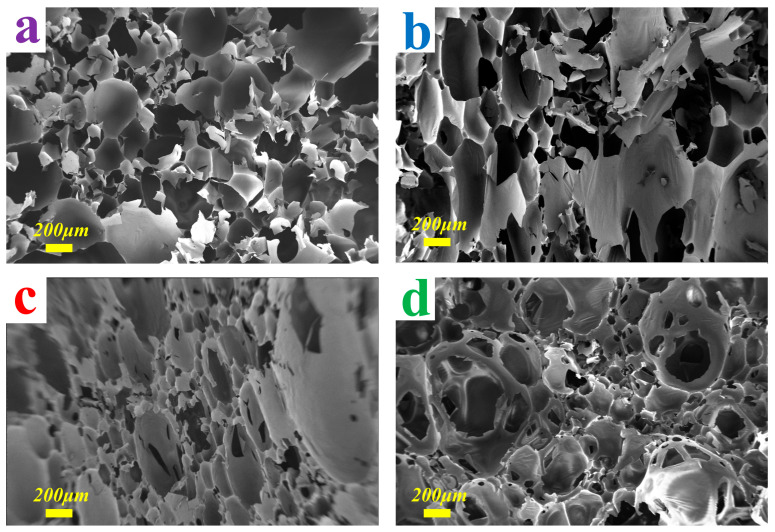
SEM micrographs at 100× magnification for the foams. (**a**) TFF; (**b**) TGF; (**c**) MTF; (**d**) STDF.

The morphological characteristics of the four types of foam samples are exhibited in Figure 3. To explore the relationship between density and pulverization ratio from the different samples, foam samples with the same density (80 kg/m^3^) were selected. From the obtained micrographs, the cells of TGF appeared denser than those of TFF, and the cells were more tightly connected to each other, which effectively confirmed that the pulverization ratio of TGF was lower than that of TFF under the same measurement range. In addition, the foam samples prepared using the new method were very noticeable by the different cell shapes, compared to the foam prepared with conventional foaming methods. The foam cells of MTF showed a very irregular morphology, presumably due to the rapid stirring, while the volume of air penetrating into the resin was varied, which caused the cells to acquire an uneven structure. This method depends upon using the difference in water addition to control the density of the foam. However, the amount of air introduced during stirring is also critical to the final foam structure, regarding bubbling. The STDF showed a distinctive vesicular structure with fully half-open pores, which had rarely been seen in previous experiments. The main reason for the formation of these vesicles was the constant movement of water vapor under steam conditions, which impinged on the cells, resulting in the formation of cavity connections between the cells [20]. As a result, the cells did not mature completely to form closed pores between them, and therefore exhibited a very significant half-open pore shape. The special preparation method of STDF also ensured that the density of the foam did not encounter large variations, compared to the density of conventional foams. However, this method greatly reduces the pulverization ratio, a phenomenon that has never been seen in previous experiments, which is of great interest.

**Figure 4:**
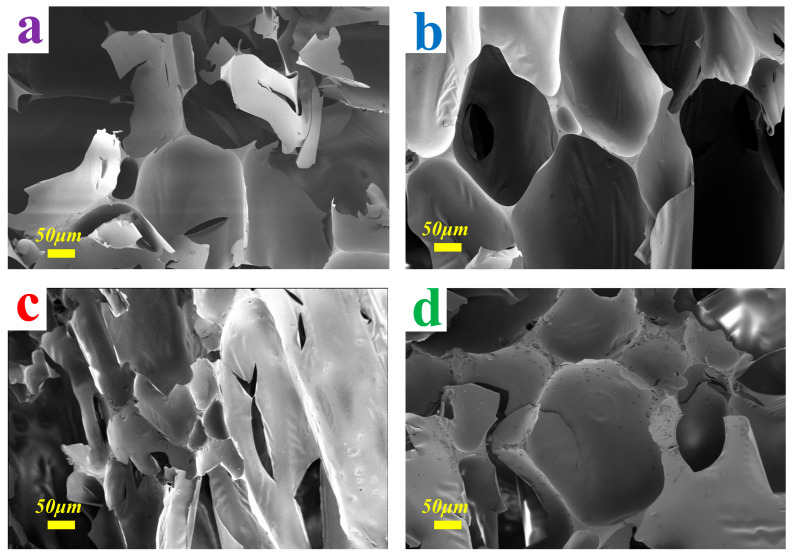
SEM micrographs at 400× magnification for the foams. (**a**) TFF; (**b**) TGF; (**c**) MTF; (**d**) STDF.

Furthermore, the magnification of the SEM has been further adjusted in Figure 4, so as to observe the structure of the cell structure more clearly. With an SEM plot of 0.4 kX, we obtained some more convincing details. The higher magnification allowed the connection patterns between the bubble pores to be observed more clearly, and it was hypothesized that the microscopic reason for the different bubble pulverization ratio was the thickness of the cell wall. As can be seen more clearly in Figure 4a,b, the cell wall thicknesses of the two samples are closer to each other, confirming on a physical level that the range of pulverization ratio of the two substances is closer [26]. The cell of MTF showed the same morphology between the different cells, leading to reasonable values of pulverization ratio and better stability. From Figure 4d, the STDF shows a very low pulverization ratio. The width of the cell walls was clearly larger than those of the other three samples and the inter-connectivity was more dense, both of which ensure that the foam remains better stabilized in spite of external factors [27]. Presumably this is the reason for which STDF exhibited such a low pulverization ratio compared to conventional TF.

**Figure 5:**
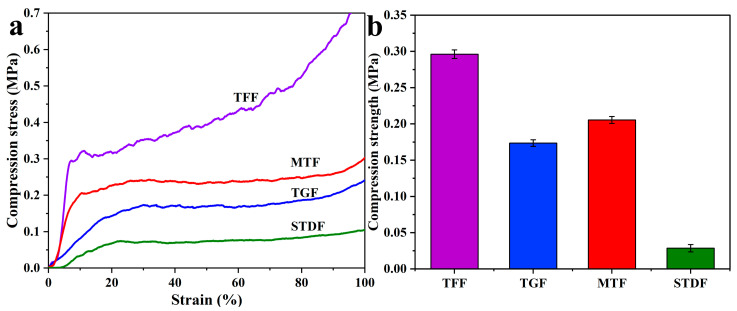
(**a**) Stress–strain curves for compression of TFF, TGF, MTF and STDF; (**b**) Compression strength of TFF, TGF, MTF and STDF.

From Figure 5a,b, to further recognize the differences between the compressive strength values of the prepared foams via different preparation methods, it can be implied that TFF (prepared by formaldehyde) had the best compression strength, mainly due to the uniformity of the foam cells, which helped to distribute the compression force very well, then achieved force equilibrium [4]. This phenomenon was very clearly demonstrated in the SEM, where the reduced uniformity of bubble cells was an important factor in the inferiority of TGF compared to TFF [6]. In the case of MTF, the rapid agitation of the bubbles made it easier to form a homogeneous bubbled liquid phase, which led to a uniform distribution of bubble cells after curing [21]. As a result, the foams prepared in this way also presented good compression strength. In the case of STDF, it acquired half-open cell morphology for all its cells, a phenomenon that could be clearly observed by SEM [20]. This was an obvious disadvantage in terms of compressive strength, as these interacting channels on the cell wall of the foam structure could destroy the integrity of the overall structure. The carrying capacity of the foam cell was significantly reduced, caused by those channels, although the excellent cross-linking of the cell walls resulted in a lower comminution ratio, with no significant advantage in terms of compressive strength (Figure 5a).

**Figure 6:**
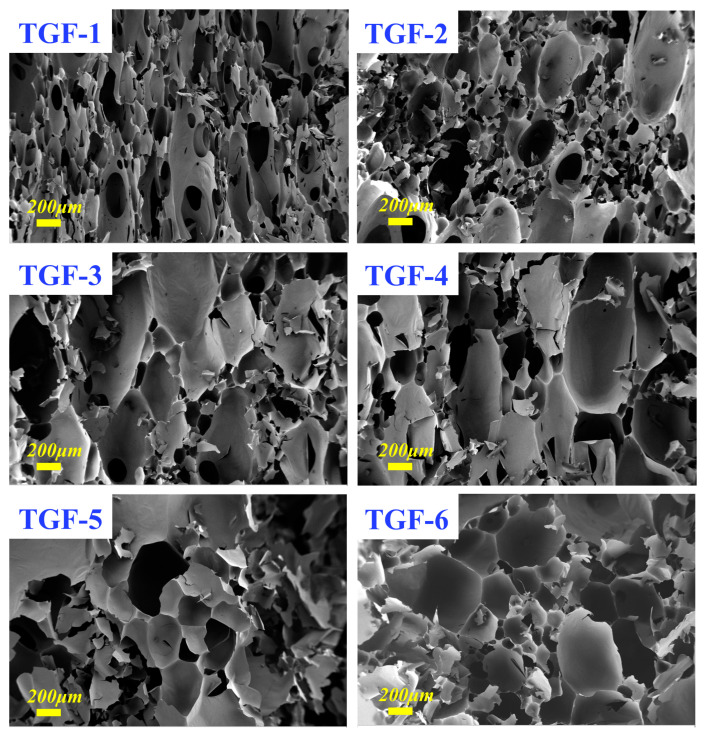
SEM micrographs at 100× magnification for the foams. (TGF1–6): TGF foams prepared with different doses of blowing agent.

A representative sample was selected to further investigate the relationship between density and pulverization ratio. Glutaraldehyde is an appropriate cross-linker compared with other aldehydes, especially in the traditional tannin-furanic foam formulation. Foams with different densities were prepared by adjusting the amount of foaming agent (ether-based, with doses ranging from 2 g to 7 g). TGF-(1–6) are foam samples with different densities. The corresponding SEM micrographs of TGF-(1–6) are shown in Figure 6, which evidently shows the morphology of the foam structure. Meanwhile, the cell size for these foams revealed an increased phenomenon from sample TGF-1 to TGF-6, meaning that the density of the foam samples will display a decrease in this trend, which is in line with the data for the original density evaluation. In addition, the SEM micrographs demonstrated that the structure of the foam gradually changed from very dense small cells to sparsely perforated cells. The visual observation of the foaming deformation also indicates that, the higher the amount of foaming agent added during preparation, the larger the foaming volume. These phenomena present direct evidence that the dose of blowing agent has a very important influence on the density of the foam [24].

**Figure 7:**
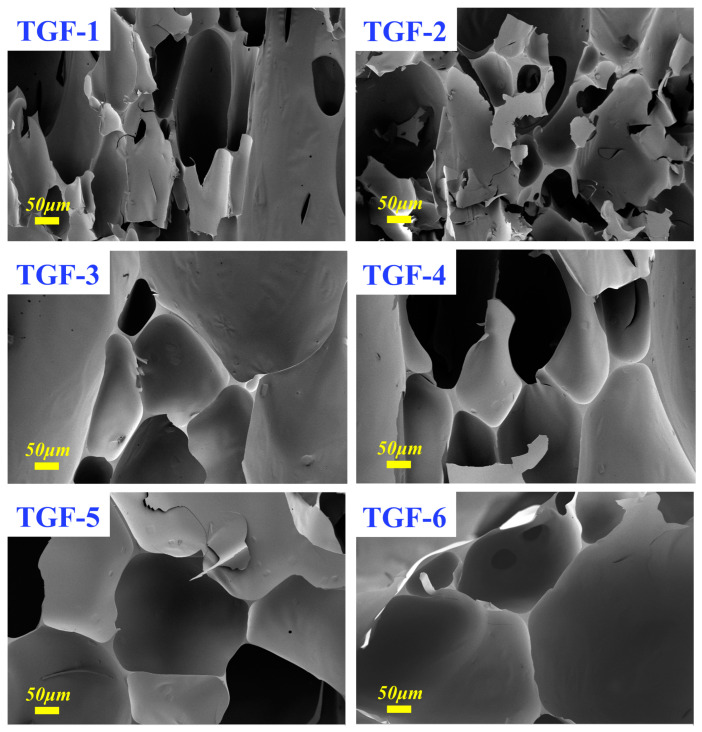
SEM micrographs at 400× magnification for the foams. (TGF1–6): GTF foams prepared with different doses of blowing agent.

In addition, to discover more interesting details, SEM images were acquired at higher magnification (400×), as demonstrated in Figure 7. The increase in the number of pores could be more conspicuously observed and the cell wall tended to become progressively thinner. Presumably based on the original data, this phenomenon could be explained by the fact that, the thicker the cell walls of the foam, the stronger the cross-linking between the substances, which gives rise to a lower value of pulverization ratio. The thicker cell walls provided a more stable cross-linked structure that was less susceptible to external damage [16]. For TGF-6, this could be explained by its large pores and thin cell walls leading to its high pulverization ratio. From Figure 4, we can similarly observe that all cell walls were smooth with no cracks detected, which could be explained by the fact that it provided a good environment for the structure of the TGF to be connected and at the same time had a practical and effective strengthening effect that is supposed to be responsible for the increase of the pulverization ratio. In addition, the triangular areas formed in the foam during foaming contributed even more to the stability of the foam structure and contributed effectively to reducing the pulverization ratio of the foam.

**Figure 8:**
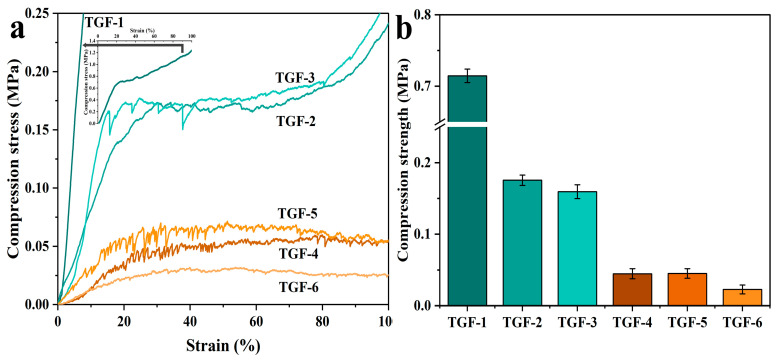
(**a**) Stress–strain curves for compression of TGF-(1–6); (**b**) Compression strength of TGF-(1–6).

The TGF-(1–6), which were prepared in the same way, were then tested for compression strength. The corresponding results are listed in Figure 8a,b. A remarkable phenomenon is that the strength of TGF-1 was 0.71 MPa, which is the highest among all the foams. This also corresponds to the result that TGF-1 had the highest density and the best structural solidness. The dense foam structure was better able to resist external pressure, which caused its high compression strength [17]. The bubble pores of the foam gradually increased, and the compressive strength decreased as the density decreased. The samples of TGF-3 and TGF-2 showed a significant decrease compared to TGF-1; however, the difference between 0.17 MPa and 0.16 MPa is small for a standard tannin-furanic based foam of 0.15 MPa. Interestingly, there was a significant, if not precipitous, reduction in the compression strength of TGF-4. The previous section illustrated that the obvious inflection point for density occurs at TGF-4, as does the inflection point for pulverization ratio. In addition, once again we found through the pressure diagram that the critical change in stress-strain behavior for compression of TGF occurred at TGF-4. It follows that the amount of foaming agent in the case of TGF-4 is crucial to the study of foams. Subsequent experiments carried out on TGF-5 and TGF-6 did not corroborate very good values.

## 4. Conclusions

A correlation between the density and pulverization ratio of foams can be explored and basic data can be analyzed on foam samples prepared by four common different methods, each of which was adopted to give the same density for the foam. These basic experiments can be helpful in predicting a relationship between the density and pulverization ratio for the foams. This can be confirmed in a non-linear fitting equation with specific values obtained for the inflection point. Digital images of the foams at different magnifications can also offer better understanding of the reasons leading to high or low pulverization ratios of the foam, which helps to clearly comprehend the effect of the difference in density in the pulverization ratio of the foams. The factors affecting the density and pulverization ratio, as well as the differences between them, can be explored more deeply by comparing groups against each other while comparing the different densities within the same group. This strategy can offer an innovative use of fitted curves, where the different densities of the foam correspond to a specific value of the pulverization ratio; therefore, the density of the foam showed a strict inverse relationship. The analysis of all types of foams in terms of compression strength can provide supporting information on production differences concerning stability for foams prepared in different ways. In addition, the non-linear fitted regression curves can indicate inflection points for the different formulations, which is considered a very interesting stage for the next step in foam research to help researchers prepare suitable foams with optimized characteristics.

## Figures and Tables

**Table 1:** Formulations of various tannin foams.

Types	Principal Substance						Density-Adjusted Substance	Ref.
	Basic Substance		Cross-Linker		Additive	Catalyst	Blowing Agent	
TFF	Tannin	Furfuryl alcohol	Formaldehyde	-	Tween-80	p-TSA	Ether	[4]
TGF			-	Glutaraldehyde				[6]
MTF			Formaldehyde	-			Water	[21]
STDF			-	-				[20]

## Data Availability

Not applicable.
